# A Distributed Method for Self-Calibration of Magnetoresistive Angular Position Sensor within a Servo System

**DOI:** 10.3390/s22165974

**Published:** 2022-08-10

**Authors:** Vladimir Čeperković, Vladimir Rajović, Milan Prokin

**Affiliations:** Department of Electronics and Digital Systems, School of Electrical Engineering, University of Belgrade, Bulevar Kralja Aleksandra 73, 11120 Belgrade, Serbia

**Keywords:** magnetoresistive sensors, self-calibration, distributed systems, servo system, angle measurement

## Abstract

Magnetoresistive angle position sensors are, beside Hall effect sensors, especially suitable for usage within servo systems due to their reliability, longevity, and resilience to unfavorable environmental conditions. The proposed distributed method for self-calibration of magnetoresistive angular position sensor uses the data collected during the highest allowed speed shaft movement for the identification of the measurement process model parameters. Data acquisition and initial data processing have been realized as a part of the control process of the servo system, whereas the identification of the model parameters is a service of an application server. The method of minimizing of the sum of algebraic distances of the sensor readings and the parametrized model is employed for the identification of parameters of linear compensation, whereas the average shaft rotation speed has been used as a high precision reference for the identification of parameters of harmonic compensation. The proposed method, in addition to a fast convergence, provides for the increase in measurement accuracy for an order of magnitude. Experimentally obtained measurement uncertainty was better than 0.5°, with the residual variance less than 0.02°, comparable to the sensor resolution.

## 1. Introduction

Precise servo systems have a wide range of applications in the automotive industry, being used for vehicle steering, the flow control of the air–fuel mixture in an internal combustion engine, and for the control of control surfaces for air routing in ventilation systems. They are expected to function reliably [1] in an extremely unfavorable operating environment, within a wide range of environment temperatures and in the presence of vibrations, dirt, and moisture. The selection of an appropriate mechanical angle sensor is critical for the accomplishment of these requirements.

A high-temperature stability and mechanical robustness [2] have contributed to the popularity of magnetoresistive (MR) angular position sensors based on the utilization of anisotropic magnetoresistive (AMR), giant magnetoresistive (GMR), or tunnel magnetoresistive (TMR) effects. For these sensors, a permanent magnet attached to the shaft creates a magnetic field, whose orientation is measured by means of MR elements. The key aspect of the application of MR sensors is the correct alignment of the magnet and the sensor, since even small deviations cause unacceptably high measurement errors. Therefore, in the presence of inevitable mechanical tolerances, the utilization of end-of-line (EOL) calibration [3,4,5] is mandatory in order to achieve a desired accuracy.

The utilization of magnetoresistive sensors is particularly demanding for the measurement of the angular position of high-pressure regulation valves. The magnet is housed in a stainless-steel capsule at a relatively long distance from the sensor and with a significant error of alignment of the rotation axis, the magnet, and the sensor. Because of that, the magnetic field intensity is low at the sensor, with particularly noticeable parasitic components; in this case, MR sensors are the transducers of choice due to an order of 50 times higher sensitivity, wider bandwidth, and less noise than Hall sensors. At the same time, the EOL calibration cannot be implemented since the shaft is not accessible; even if it were so, the calibration would be time consuming and expensive and the sensor would go out of calibration after a relatively short time of exploitation due to temperature drift and mechanical vibrations. This incurs the essential need for and the importance of online self-calibration.

Possible reasons for nonlinear measurement errors were analyzed in [6]. Although the study deals with the Hall sensors with a magnetic flux concentrator only, the results are applicable to the MR angular sensors as well. The influence of a permanent magnet characteristic and mechanical tolerances to the measurement error of GMR angular position sensor were studied in [7,8]. A magnet and a sensor must be carefully aligned during the installation; otherwise, the accuracy would be significantly reduced. Specifically, the MR sensor errors are periodical, with a 180° period.

A method for self-calibration of a resolver in a speed servo system, based on a linear model of the measurement process, was proposed in [9]. The gradient algorithm for the identification of the linear compensation parameters was employed; so, the mean square deviation of the compensated signal from the unit circle was minimized. Unfortunately, this method utilizes an extended observer of the angular position as a reference, thus limiting the accuracy of the self-calibration. Another disadvantage of the method is a relatively slow convergence, estimated in [9] to be several tens of rotation periods long. Another method [10] utilizes the model-based automatic search algorithm (MASA) for the resolver self-calibration, employing a polynomial nonlinear model of measurements and a back-propagation gradient method for optimization.

The self-calibration of AMR sensors described in [11] applies a simplified linear sensor model, whose parameters are obtained by the utilization of a gradient algorithm. It was shown that the residual error in this case depends significantly on the initial estimation of the model parameters. The problem of the measurement offset was solved in [12] by the utilization of a simple system of equations, with the assumptions that the measurement system noise is negligible, the sensitivities of both bridges are identical, and that there are no other sources of errors.

A solution to the problem of the measurement sensitivity to the relative positioning of the magnet axis and sensor was presented in [5] in a form suitable for the EOL calibration. A sensor matrix was used together with the theoretical model of the magnetic field in order to determine the polynomial compensation function. The identical sensor matrix principle was utilized in [13] as well, in order to identify and compensate for the influence of an external magnetic field. An alternative approach, analyzed in [14], consists of the utilization of the physical protection made of a material with a high magnetic permeability. Nevertheless, the proposed concept has not been practically implemented due to difficulties in the production of such a protection as well as the limited space available for the housing of a measurement system.

One of the methods for the compensation of the MR angular position sensor, applicable in the case when EOL calibration cannot be undertaken, was presented in [15]. The compensation of the measurement error consists of the linear compensation of the magnetic field vector measurement error and the harmonic compensation of the angular position measurement error. The self-calibration method utilizes the data acquired during a maximum allowed speed movement for the identification of parameters of the measurement process model. The identification is undertaken every time the shaft finishes a revolution. The least squares method has been used for the identification of parameters of linear compensation, whereas the average shaft rotation speed is utilized as a high-precision reference for the identification of the parameters of harmonic compensation. Unfortunately, the presented method is particularly computationally demanding; therefore, it is not suitable for a practical direct implementation.

Automotive peripheral devices are often based on embedded 8-bit microcontrollers (MCU), for example [16], because of their high reliability and low cost. At the same time, the insufficient computational power of the MCUs have limited implementation of complex methods of self-calibration. However, with the emergence of the fourth generation of the electrical/electronic (E/E) automotive platforms [17], with a dedicated domain control unit (DCU) based on the AUTOSAR standard [18], the available computing capacities are significantly increased. Namely, such a platform includes at least one application server (AS) for the execution of computationally demanding services, making it possible to realize a self-calibration method in a distributed form.

However, a disadvantage of (self-)calibration is that a change of compensation parameters causes a momentary change of the measured position, possibly leading to an oscillatory transient process of the servo system. This was overcome in [15] by invoking the procedure for the calculation of the parameters, and their change, only when the system is in its referent position, which is calibration invariant. However, such a method would not be applicable in a distributed scenario.

The aim of this paper was the development of a new method for a distributed self-calibration of MR angular position sensors in servo systems, wherein the compensation of the measurement error is tuned on the basis of the identified parameters of the sensor model. The tuning is performed at the times when the shaft finishes a revolution while rotating at a maximum speed. The least squares method was used for the identification of the parameters of the model. The method was adapted to a distributed realization within a heterogeneous infrastructure of an automotive communication network. It was shown that the utilization of the proposed self-calibration method increased the accuracy of the measurement for an order of magnitude. Measurement uncertainty less than 0.5°, comparable to the results of modern EOL calibration procedures, were experimentally confirmed.

The paper is structured as follows. The self-calibration method, including the measurement process and the procedure for the identification of the model parameters, is presented in Section 2. The procedure of distributed processing adapted to a heterogeneous infrastructure of an automotive communication network is presented in Section 3. Experimental verification of the measurement method is presented in Section 4. Finally, Section 5 contains the conclusions of this paper.

## 2. The Self-Calibration Method

**Figure 1:**
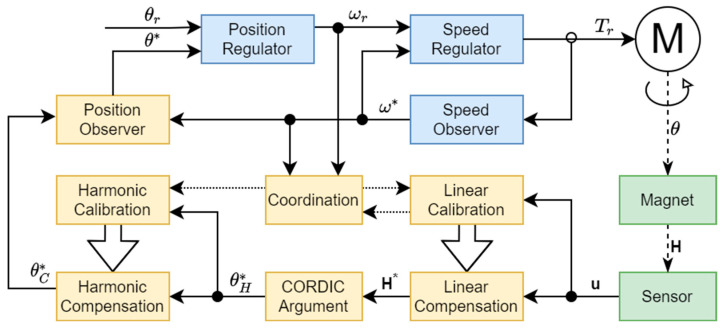
The structure of a servo system with the angular position measurement system.

The structure of a servo system with the angular position measurement system is shown in Figure 1. A permanent magnet is attached to a motor shaft; so, the magnetic field and the shaft rotate synchronously. There is an MR angular sensor mounted below the magnet, detecting the magnetic field H in the plane of the sensor. The result of the detection, after amplification and A/D conversion, is a two-dimensional input signal u that is used for further processing by the application of the inverse sensor model in order to calculate the result of the angular position measurement $\theta^{*}$. At the same time, the input signal u is used for the self-calibration process in which the parameters of the sensor model are identified.

The sensor inverse model consists of four sequential processes. Firstly, by linear compensation, the systematic errors are eliminated and the result of the magnetic field vector $H^{*}$ measurement is calculated. Then, by utilization of the CORDIC (coordinate rotation digital computer) algorithm for the calculation of a vector argument, the result of the magnetic field angle $\theta_{H}^{*}$ measurement is obtained. In the next step, by the application of harmonic compensation, the deviation between the angular position and the magnetic field angle is corrected and the compensated measured angular position $\theta_{C}^{*}$ is calculated. Finally, by filtering in the position observer, the result of the measurement of the angular position $\theta^{*}$ is obtained.

The control subsystem of the servo system consists of a cascade connection of the position regulator and the speed regulator. The speed regulator is based on the observer of the rotation speed $\omega^{*}$; therefore, it does not use the result of the measurement of the angular position at high speeds, whereas the possible use of the result at low rotation speeds does not affect the application of the proposed method. At the same time, the set speed $\omega_{r}$ is continuously changed in accordance with the control law implemented in the position regulator, depending on the difference between the set angular position $\theta_{r}$ and the result of the measurement of the angular position $\theta^{*}$. When the difference becomes large enough, the position regulator enters the saturation mode, where the regulator output is constant, equal to the maximum allowed speed. Thus, the measurement of the angular position does not affect the set rotation speed, breaking the feedback loop in effect. Consequently, the rotation speed, the angular position, and the input signal u are all independent from the process of measurement of the angular position, making it possible to use them for the identification of the parameters of the sensor model without disturbing the control loop.

The steady state in the saturation mode of the position regulator, while the shaft of the motor rotates at a constant speed, is of a special interest. Namely, it is possible to measure the average shaft speed with a high accuracy by measuring the rotation period, and the average speed can be used as an internal, high-precision reference. Thus, the self-calibration procedure is executed in the steady state, during the saturation mode of the position regulator only, when the shaft of the motor rotates at a maximum allowed speed. If the estimated or the set speed decreases for any reason, the procedure is aborted. The estimation of the model parameters is performed at the times when the shaft finishes a revolution. This procedure ensures a representativeness of the sample and the stability of the estimated model parameters.

### 2.1. The Process of the Magnetic Field Angle Measurement

**Figure 2:**
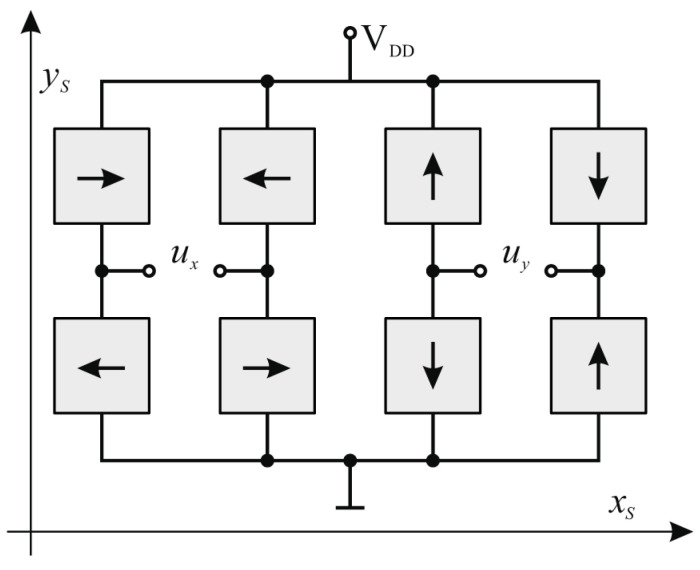
The structure of the measurement bridges within an MR angular sensor.

A magnetoresistive angular position sensor, whose structure is shown in Figure 2, measures the projection of the magnetic field vector in the plane of the sensor. The sensor consists of eight MR elements, connected within two measurement bridges. The reference vectors of the four MR elements connected in the first bridge ($u_{x}$) are aligned to the $x_{S}$ axis of the sensor, whereas the reference vectors of the four MR elements in the second bridge ($u_{y}$) are aligned to $y_{S}$-axis of the sensor.

Due to the limitations of the photolithographic process, the $x_{S}$ axis and $y_{S}$ axis of a real sensor are only approximately orthogonal. Without the loss of generality, it is assumed that the sensor’s $y_{S}$ axis is the reference axis, whereas the sensor’s $x_{S}$ axis differs from the ideal *x*-axis for an angle ϕ clockwise. Then, the linear model of an MR angle sensor is described as

(1) $$\begin{array}{c} u={\left[\begin{array}{cc} u_{x} & u_{y} \end{array}\right]}^{T}=G\cdot H+o,\\ G=\left[\begin{array}{cc} k_{x}\cdot \cos\left(\phi \right) & -k_{x}\cdot \sin\left(\phi \right)\\ 0 & k_{y} \end{array}\right]. \end{array}$$

The parameter o models the residual offset of the analog interface, the inherent offset of the measurement bridges [2,3,12], and the external magnetic field [7], whereas parameters $k_{x}$ and $k_{y}$ model different sensitivities of the bridges X and Y [2,11]. Since the matrix G is a regular matrix, the transfer function of the linear compensation of the input signal u is obtained by the inversion of the linear model Equation (1)

(2) $$H^{*}=G^{-1}\cdot \left(u-\;o\right).$$

Finally, the result of the measurement of the magnetic field angle $\theta_{H}^{*}$ is the argument of the measured magnetic field vector Equation (2)

(3) $$\theta_{H}^{*}=m^{-1}\cdot \arg\left\{G^{-1}\cdot \left(u-o\right)\right\}.$$

The integer geometric constant *m* depends on the type of the MR sensor and the number of poles p of the employed permanent magnet, equaling 2p if it is an AMR sensor and p if it is a TMR or GMR. It should be noted that the multiplication by this constant must be performed within the argument calculation because the resulting angle should be unwrapped *m* times.

### 2.2. Determination of the Linear Compensation Parameters

The trace of the signal u from the model Equation (1) outlines an ellipse, which could be presented in an implicit form, using the algebraic distance function f(u;q) with parameters vector q:

(4) $$\begin{array}{c} f(u;q)=\left[\begin{array}{cccccc} u_{x}^{2} & u_{x}u_{y} & u_{y}^{2} & u_{x} & u_{y} & 1 \end{array}\right]\cdot q=0,\\ q={\left[\begin{array}{cccccc} a & 2b & c & 2d & 2f & g \end{array}\right]}^{T},\\ ac-b^{2}>0. \end{array}$$

One can observe that the intensity of the parameters vector q does not affect Equation (4); therefore, it could be arbitrarily chosen so the expression $ac-b^{2}$ has a positive set value. By the adoption of the constant set value 0.25, suitably chosen so the resulting method has the smallest number of calculations, there arises an additional condition that changes the inequality from Equation (4):


(5) $$ac-b^{2}=0.25$$

The optimal parameters vector q minimizes the sum of the squares of the algebraic distances f(u;q) for a known sequence of input signal measurements u[i], i=1…N. In order to determine its value, a modification of the direct method for the determination of an ellipse equation [19,20] could be employed. To accomplish this, an auxiliary vector x[i], defined as a function of the input signal u[i], is introduced:

(6) $$x={\left[\begin{array}{cccccc} u_{x}^{2} & u_{x}u_{y} & u_{y}^{2} & u_{x} & u_{y} & 1 \end{array}\right]}^{T}.$$

Let the samples’ matrix D contain all the vectors x[i] for a known measurements’ sequence:

(7) $$D={\left[\begin{array}{cccc} x[1] & x[2] & \cdots & x[N] \end{array}\right]}^{T}.$$

The sum of the squares of algebraic distances f(u;q) for a known sequence of input signal measurements u[i] can be represented in a matrix form using the measurements’ matrix D:

(8) $$\sum_{i=1}^{N}f(u_{i};q)=q^{T}\cdot D^{T}\cdot D\cdot q=q^{T}\cdot S\cdot q,$$

where S is the covariance matrix of vector x, of size 6 x 6, which could be expressed as:

(9) $$S=D^{T}\cdot D=\sum_{i=1}^{N}x\cdot x^{T}.$$

The condition Equation (5) can be presented in the vector form:

(10) $$q^{T}\cdot C\cdot q=0.25,$$

where condition matrix C is a constant square block matrix of size 6 × 6, given by:

(11) $$C=\left[\begin{array}{cc} C_{1} & 0\\ 0 & 0 \end{array}\right],C_{1}=\left[\begin{array}{ccc} 0 & 0 & 0.5\\ 0 & -0.25 & 0\\ 0.5 & 0 & 0 \end{array}\right].$$

The Langrage multiplier corresponding, given the condition Equation (10), to the criterion Equation (8) is, according to [21], expressed as:

(12) $$ℒ=q^{T}\cdot S\cdot q-\lambda \cdot \left(q^{T}\cdot C\cdot q-0.25\right).$$

After equaling the derivative of the Lagrange multiplier with zero, the condition for the minimization of the criterion Equation (8) given the condition Equation (10) is obtained:

(13) $$\begin{array}{c} \nabla_{q}\left(ℒ\right)=2\left(S\cdot q-\lambda \cdot C\cdot q\right)=0,\\ S\cdot q=\lambda \cdot C\cdot q. \end{array}$$

The solution Equation (13) is the generalized eigenvector of the matrix pair (S,C), corresponding to a maximum generalized eigenvalue. However, since the matrix C is a singular matrix and the matrix S is an almost singular matrix, a direct solving of Equation (13) is numerically unstable. Instead, the principle presented in [19] will be used. Thus, the matrix S can be represented as a block matrix of square matrices $S_{1}$, $S_{2}$, and $S_{3}$ of size 3 × 3, and the parameters vector q can be represented as a block vector built of vectors $q_{1}$ and $q_{2}$ of length 3:

(14) $$\begin{array}{c} S=[\begin{array}{cc} S_{1} & S_{2}\\ S_{2}^{T} & S_{3} \end{array}], \end{array}\begin{array}{c} q=\left[\begin{array}{c} q_{1}\\ q_{2} \end{array}\right]. \end{array}$$

The expression Equation (13) can now be presented in a block form:

(15) $$\left[\begin{array}{cc} S_{1} & S_{2}\\ S_{2}^{T} & S_{3} \end{array}\right]\cdot \left[\begin{array}{c} q_{1}\\ q_{2} \end{array}\right]=\lambda \cdot \left[\begin{array}{cc} C_{1} & 0\\ 0 & 0 \end{array}\right]\cdot \left[\begin{array}{c} q_{1}\\ q_{2} \end{array}\right],$$

that is in the form of a system of two matrix equations:

(16) $$S_{1}\cdot q_{1}+S_{2}\cdot q_{2}=\lambda \cdot C_{1}\cdot q_{1},$$

(17) $$S_{2}^{T}\cdot q_{1}+S_{3}\cdot q_{2}=0.$$

One could note that the matrix $S_{3}$ is singular only if there is a linear dependence between the coordinates of input points, not being the case here. Therefore, Equation (17) can be transformed:

(18) $$q_{2}=-S_{3}^{-1}\cdot S_{2}^{T}\cdot q_{1}.$$

After substitution of Equation (18) in Equation (16), there is

(19) $$S_{1}\cdot q_{1}-S_{2}\cdot S_{3}^{-1}\cdot S_{2}^{T}\cdot q_{1}=\lambda \cdot C_{1}\cdot q_{1},$$

and after solving and extracting the auxiliary matrix M:

(20) $$\begin{array}{c} M=C_{1}^{-1}\cdot \left(S_{1}-S_{2}\cdot S_{3}^{-1}\cdot S_{2}^{T}\right),\\ M\cdot q_{1}=\lambda \cdot q_{1}. \end{array}$$

The optimal value of the vector $q_{1}$ is obtained as the eigenvector of matrix M with the maximum eigenvalue; the value of $q_{2}$ is then obtained from Equation (18). The optimal value of vector q is finally obtained by combining the two vectors.

On the other hand, the ellipse Equation (4) can be presented in canonical form using a linear transformation of coordinates:

(21) $$u=R\left(\phi_{1}\right)\cdot \left[\begin{array}{cc} k_{x} & 0\\ 0 & k_{y} \end{array}\right]\cdot R\left(\phi_{0}\right)\cdot H+\left[\begin{array}{c} x_{0}\\ y_{0} \end{array}\right],$$

where R(α) is the rotation matrix:

(22) $$R\left(\alpha \right)=\left[\begin{array}{cc} \cos\left(\alpha \right) & -\sin\left(\alpha \right)\\ \sin\left(\alpha \right) & \cos\left(\alpha \right) \end{array}\right]$$

After the substitution of Equation (21) in Equation (4) and solving, the parameters of a canonical ellipse form in the function of the known ellipse parameters q are obtained:

(23) $$\begin{array}{c} \Delta =\sqrt{{\left(a-c\right)}^{2}+4\cdot b^{2}},\\ k_{x,y}=-\sqrt{2\cdot \left(4\cdot a\cdot f^{2}+4\cdot c\cdot d^{2}-8\cdot b\cdot d\cdot f-g\right)\cdot \left(a+c\mp \Delta \right)},\\ \begin{array}{cc} x_{0}=4\cdot \left(b\cdot f-c\cdot d\right), & y_{0}=4\cdot \left(b\cdot d-a\cdot f\right),\\ \phi_{0}=\arctan\left(-\frac{k_{x}\cdot \sin\left(\phi_{1}\right)}{k_{y}\cdot \cos\left(\phi_{1}\right)}\right), & \phi_{1}=\arctan\left(\frac{c-a-\Delta }{2\cdot b}\right). \end{array} \end{array}$$

Finally, after the comparison of Equation (23) and the linear model Equation (2), the parameters of the linear compensation are obtained:

(24) $$\begin{array}{c} o={\left[\begin{array}{cc} x_{0} & y_{0} \end{array}\right]}^{T},\\ G^{-1}=R\left(\phi_{0}\right)\cdot \left[\begin{array}{cc} k_{x}^{-1} & 0\\ 0 & k_{y}^{-1} \end{array}\right]\cdot R\left(-\phi_{1}\right). \end{array}$$

The precision of the parameters in Equation (24) does not depend on the precision of the rotation speed, that is, the precision of the speed regulator and the speed observer, a consequence of the usage of the implicit ellipse model in Equation (4). Therefore, for the correct identification, it is sufficient that the matrix D contains representative sample data enclosing the full angle.

### 2.3. The Process of Measurement of the Angular Position

The results presented in [6,7,8] show that a nonlinear measurement error, arising as a consequence of the characteristics of permanent magnets and mechanical tolerances of the system, is a slowly changing periodical function of the angular position. Therefore, a harmonic model of the angular position θ dependent on the magnetic field angle $\theta_{H}$ is selected:

(25) $$\theta =\theta_{H}-h_{0}-h\left(\theta_{H}\right),$$

where $h_{0}$ is a constant offset and $h\left(\theta_{H}\right)$ is a harmonic corrector given as:

(26) $$h\left(\theta_{H}\right)=\sum_{k=1}^{n}\left[a_{k}\cos\left(k\cdot \theta_{H}\right)+b_{k}\sin\left(k\cdot \theta_{H}\right)\right].$$

In the corrector Equation (26), $a_{k}$ and $b_{k}$ are the model parameters and n is the order of the harmonic corrector. This corrector is a consequence of magnet polarization and the misalignments of the axes of rotation, the magnet, the sensor, and the inclination of the sensor in reference to the magnet. The amplitudes of the harmonics decrease fast; so, a satisfactory precision of the model was accomplished with the first two harmonics already.

The measured compensated angular position $\theta_{C}^{*}$ is obtained by the application of the models’ Equation (25) and Equation (26) to the measured magnetic field angle $\theta_{H}^{*}$ from Equation (3):

(27) $$\theta_{C}^{*}=\theta_{H}^{*}-h_{0}-h\left(\theta_{H}^{*}\right).$$

The result of the harmonic compensation Equation (27) is filtered in the angular position observer, described in z-domain as:

(28) $$\theta^{*}=\frac{K\cdot \theta_{C}^{*}+\omega^{*}\cdot T_{S}}{z-\left(1-K\right)},$$

where $\theta^{*}$ is the measured angular position, K is the gain of the observer error, $\omega^{*}$ is the estimation of the current rotation speed, and $T_{S}$ is the sampling period. The angular position observer is necessary for the implementation of the distributed measurement calibration when there is a possibility of an asynchronous change of the system parameters. Namely, a change in parameters of either linear or harmonic calibration causes an instant change of the compensated angular position $\theta_{C}^{*}$, and, without the filtering provided by the observer, the position regulation loop could be negatively affected.

### 2.4. Determination of the Harmonic Compensation Parameters

The parameters of the harmonic model are identified by the comparison of the reference angle with the sequence of the measured values of the magnetic field angle $\theta_{H}^{*}\left[i\right]$. At the same time, when the rotation speed is constant, the reference angle is:

(29) $$\theta_{ref}\left(i\right)=\theta_{1}\left(i\right)+\theta_{0},$$

where $\theta_{1}$ is the linear component of the reference angle given as:

(30) $$\theta_{1}\left(i\right)=\frac{2\pi }{N}\left(i-1\right),$$

and $\theta_{0}$ is the initial value of the reference angle, which is not a priori known. Since it is adopted that the $y_{S}$ axis of the sensor is the reference axis, the initial value of the reference angle is determined on the basis of the passage of the measured value of the magnetic field angle $\theta_{H}^{*}\left[i\right]$ through zero. The error of the reference angle Equation (29) is the direct consequence of the deviation of the current speed from the average speed. As both the speed regulator and the observer are designed to suppress the error of speed in the low-frequency part of spectrum, the error of reference in the stationary state contains high-frequency components only.

After Equation (25) is applied to the reference angles Equation (29) and solved for the harmonic corrector, the following is obtained:

(31) $$h_{0}+h\left(\theta_{H}^{*}\left[i\right]\right)=\theta_{H}^{*}\left[i\right]-\theta_{ref}\left(i\right).$$

The optimal coefficients of the harmonic corrector $h_{0}$, $a_{k}$ and $b_{k}$, can be determined from Equation (31) by the means of linear regression for a known sequence of measured values of the magnetic field angle $\theta_{H}^{*}\left[i\right]$. Unfortunately, such a method demands operations with relatively large matrices, whose order increases with the order of the corrector Equation (26). Because of that, an approximate method is developed as well, based on the observation that the maximum amplitude of the harmonic corrector Equation (26) is relatively small, less than 1.5° in practical applications, and could be neglected in the first approximation:

(32) $$h_{0}+h\left(\theta_{H}^{*}\left[i\right]\right)\approx h_{0}\to \theta_{H}^{*}\left[i\right]\approx \theta_{ref}\left(i\right)+h_{0}.$$

Since the harmonic corrector Equation (26) is a smooth function slowly changing with the argument, the application of the approximation Equation (32) to the argument of the harmonic corrector α(i) in Equation (31) will not bring up a significant error. Therefore:

(33) $$\begin{array}{c} h_{0}+h\left(\alpha \left(i\right)\right)=\theta_{H}^{*}\left[i\right]-\theta_{ref}\left(i\right),\\ \alpha \left(i\right)=\theta_{ref}\left(i\right)+h_{0}. \end{array}$$

The optimal coefficients of harmonic compensation are obtained by the utilization of linear regression in Equation (33) for a known sequence of measurements of magnetic field angle $\theta_{H}^{*}\left[i\right]$, which comes to the modified discrete Fourier transformation of difference between the reference angle Equation (29) and the measured values of magnetic field angle $\theta_{H}^{*}\left[i\right]$:

(34) $$h_{0}=\frac{1}{N}\sum_{i=1}^{N}\left(\theta_{H}^{*}[i]-\theta_{ref}\left(i\right)\right),$$

(35) $$a_{k}=\frac{2}{N}\sum_{i=1}^{N}\left(\theta_{H}^{*}[i]-\theta_{ref}\left(i\right)\right)\cdot \cos\left(k\cdot \alpha \left(i\right)\right),$$

(36) $$b_{k}=\frac{2}{N}\sum_{i=1}^{N}\left(\theta_{H}^{*}[i]-\theta_{ref}\left(i\right)\right)\cdot \sin\left(k\cdot \alpha \left(i\right)\right).$$

Unfortunately, a direct application of Equations (34)–(36) would demand knowledge of the entire sequence of the measured values of the magnetic field angle $\theta_{H}^{*}[i]$, mostly because it is needed to determine the value of the coefficient $h_{0}$ first but also because the starting reference angle $\theta_{0}$ is initially not known. Therefore, Equation (34) is transformed in order to separate the value that could be calculated independently from the reference angle $\theta_{0}$:

(37) $$h_{0}=\frac{1}{N}\sum_{i=1}^{N}\left(\theta_{H}^{*}[i]-\theta_{1}\left(i\right)-\theta_{0}\right)=F_{0}-\theta_{0},$$

where $F_{0}$ is the partial sum:

(38) $$F_{0}=\frac{1}{N}\sum_{i=1}^{N}\left(\theta_{H}^{*}[i]-\theta_{1}\left(i\right)\right).$$

After the substitution of Equations (29) and (37) in Equation (33), the argument α(i) could be expressed independently from the starting reference angle:

(39) $$\alpha \left(i\right)=\theta_{1}\left(i\right)+F_{0}.$$

Next, after the substitution of Equation (39) in Equation (35) and rearrangement, there is:

(40) $$\begin{array}{c} a_{k}=\frac{2}{N}\sum_{i=1}^{N}\left(\theta_{H}^{*}[i]-\theta_{1}\left(i\right)-\theta_{0}\right)\cdot \cos\left(k\cdot \theta_{1}\left(i\right)+k\cdot F_{0}\right)\\ =\frac{2}{N}\sum_{i=1}^{N}\left(\theta_{H}^{*}[i]-\theta_{1}\left(i\right)\right)\cdot \cos\left(k\cdot \theta_{1}\left(i\right)\right)\cdot \cos\left(k\cdot F_{0}\right)\\ -\frac{2}{N}\sum_{i=1}^{N}\left(\theta_{H}^{*}[i]-\theta_{1}\left(i\right)\right)\cdot \sin\left(k\cdot \theta_{1}\left(i\right)\right)\cdot \sin\left(k\cdot F_{0}\right)\\ =\cos\left(k\cdot F_{0}\right)\cdot \frac{2}{N}\cdot \sum_{i=1}^{N}\left(\theta_{H}^{*}[i]-\theta_{1}\left(i\right)\right)\cdot \cos\left(k\cdot \theta_{1}\left(i\right)\right)\\ -\sin\left(k\cdot F_{0}\right)\cdot \frac{2}{N}\cdot \sum_{i=1}^{N}\left(\theta_{H}^{*}[i]-\theta_{1}\left(i\right)\right)\cdot \sin\left(k\cdot \theta_{1}\left(i\right)\right). \end{array}$$

Similarly, after the substitution of Equation (39) in Equation (36) and the rearrangement, there is:

(41) $$\begin{array}{c} b_{k}=\frac{2}{N}\sum_{i=1}^{N}\left(\theta_{H}^{*}[i]-\theta_{1}\left(i\right)-\theta_{0}\right)\cdot \sin\left(k\cdot \theta_{1}\left(i\right)+k\cdot F_{0}\right)\\ =\frac{2}{N}\sum_{i=1}^{N}\left(\theta_{H}^{*}[i]-\theta_{1}\left(i\right)\right)\cdot \sin\left(k\cdot \theta_{1}\left(i\right)\right)\cdot \cos\left(k\cdot F_{0}\right)\\ +\frac{2}{N}\sum_{i=1}^{N}\left(\theta_{H}^{*}[i]-\theta_{1}\left(i\right)\right)\cdot \cos\left(k\cdot \theta_{1}\left(i\right)\right)\cdot \sin\left(k\cdot F_{0}\right)\\ =\cos\left(k\cdot F_{0}\right)\cdot \frac{2}{N}\sum_{i=1}^{N}\left(\theta_{H}^{*}[i]-\theta_{1}\left(i\right)\right)\cdot \sin\left(k\cdot \theta_{1}\left(i\right)\right)\\ +\sin\left(k\cdot F_{0}\right)\cdot \frac{2}{N}\sum_{i=1}^{N}\left(\theta_{H}^{*}[i]-\theta_{1}\left(i\right)\right)\cdot \cos\left(k\cdot \theta_{1}\left(i\right)\right). \end{array}$$

The partial sums $F_{C,k}$ and $F_{S,k}$ are identified from Equations (40) and (41) next:

(42) $$\begin{array}{c} F_{C,k}=\sum_{i=1}^{N}\left(\theta_{H}^{*}[i]-\theta_{1}\left(i\right)\right)\cdot \cos\left(k\cdot \theta_{1}\left(i\right)\right),\\ F_{S,k}=\sum_{i=1}^{N}\left(\theta_{H}^{*}[i]-\theta_{1}\left(i\right)\right)\cdot \sin\left(k\cdot \theta_{1}\left(i\right)\right). \end{array}$$

It should be noted that the partial sums from Equations (38) and (42) can be calculated incrementally, without the knowledge of the entire sequence of the measured values of the magnetic field angle $\theta_{H}^{*}[i]$. At the same time, the parameters of the model, $a_{k}$ and $b_{k}$, can be determined solely from these sums by the substitution of Equation (42) in Equations (40) and (41):

(43) $$\begin{array}{c} a_{k}=\frac{2}{N}\cdot \left(\cos\left(k\cdot F_{0}\right)\cdot F_{C,k}-\sin\left(k\cdot F_{0}\right)\cdot F_{S,k}\right),\\ b_{k}=\frac{2}{N}\cdot \left(\cos\left(k\cdot F_{0}\right)\cdot F_{S,k}+\sin\left(k\cdot F_{0}\right)\cdot F_{C,k}\right). \end{array}$$

For the determination of the coefficient $h_{0}$, it is sufficient to note that a well-tuned harmonic corrector has no influence when the measured direction of the magnetic field vector matches the reference axis. Since it is assumed that the $y_{S}$ axis of the sensor is the reference axis, the necessary condition is:

(44) $$h_{0}+h\left(0\right)=0.$$

Therefore, by solving Equation (44), the expression for the coefficient $h_{0}$, which does not include the starting value of the reference angle anymore, is obtained:

(45) $$h_{0}=-\sum_{k=1}^{N}a_{k}.$$

The developed tuning procedure is performed in two steps when the motor shaft rotates at the maximum constant speed. Firstly, the sums in Equations (38) and (42) are iteratively calculated for each measured value of the magnetic field angle. The coefficients of the harmonic compensation in Equations (43) and (45) are calculated only when the motor shaft finishes a revolution. The procedure is prematurely interrupted if the motor speed decreases for whatever reason or if the parameters of the linear compensation Equation (2) are changed.

The application of the low-order harmonic model is essentially significant for the suppression of the internal reference error, since only the low-frequency part of the spectrum is used for the identification. The reference error, therefore, behaves as an independent random noise; so, the error of the identified parameters is inversely proportional to the square root of the input sequence length N.

## 3. The Distributed Self-Calibration Method

**Figure 3:**
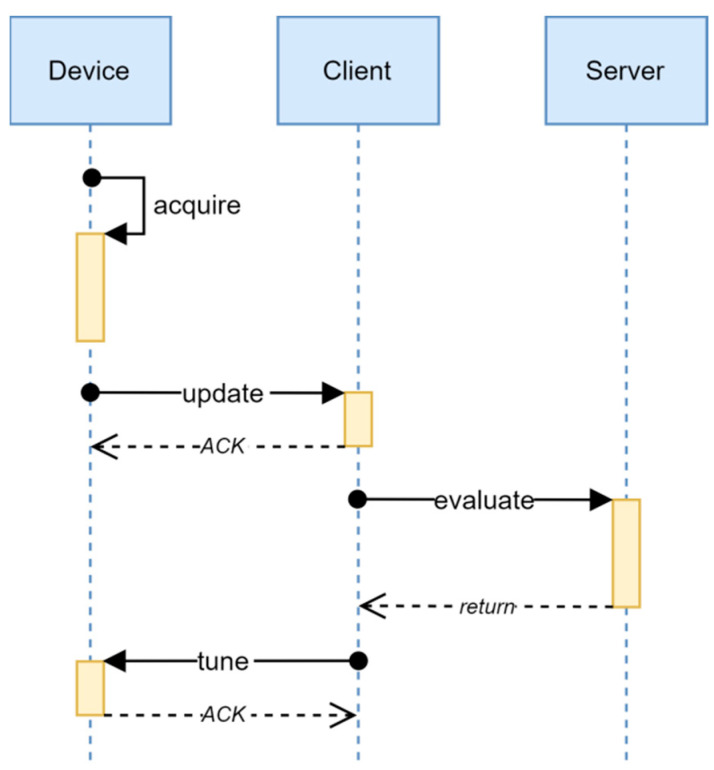
The distributed calibration method diagram.

The method for the self-calibration of the MR angular position sensors in servo systems is presented in the Section 2, consisting of independent determinations of the parameters of linear and harmonic compensations. Unfortunately, the implementation of the method for the determination of the compensation parameters requires an application of the floating-point linear algebra library, which is, in the best case, a nontrivial task, considering limited resources of an 8-bit MCU. In order to overcome this issue, without utilizing a bigger MCU, which would negatively influence the reliability and the cost of a system, the implementation of a distributed method for the determination of the compensation parameters was proposed. The time diagram of the proposed method is presented in Figure 3.

The distributed calibration method is a sequence of four independent asynchronous procedures. The execution of each successive procedure in the sequence starts only when the required computing and communication resources are freed. A sequential request number provides for following the method flow through the distributed system, even in the case when the communication infrastructure contains additional nodes, and the rejection of outdated requests. Namely, since the calibration requests are independent, it is sufficient to execute each next step in response to the request with the highest sequential request number only. All the other requests can be rejected without influencing the correctness of the result. Therefore, all the communication buffers have the capacity of one message only and the reception buffers are set to accept only messages with a higher sequential calibration number.

The execution of the distributed method begins within the device, by the procedure **acquire**, during which the needed data are collected and the calibration request is being prepared. Once a big enough data set has been processed, the calibration request is assigned a sequential number and the procedure finishes. The procedure **acquire** is realized as a part of the interrupt routine of the control algorithm, during which the data are processed incrementally for each sample of the sensor signal. The limitation of the computing time needed for the increment calculation is critical for the feasibility of the realization; thus, it is desirable to exclusively use integer operations without scaling. This is of utmost importance when 8-bit MCUs are used for the realization, since shifting of multibyte registers is a computationally expensive operation. Because of that, it is necessary that the algorithms and the data structures are adapted for such a realization.

During the procedure **update**, the calibration request is being transferred from the device to the client. It is desirable that the transmission is performed over a reliable channel since the data collection depends on external factors and the time to the next request cannot be guaranteed. However, it is sufficient to guarantee a transmission with “at least once” semantics, since the sequential request number can be used for the rejection of a repeated request.

The procedure **evaluate** is entirely executed in the background process of the application server as servicing of the Remote Procedure Call (RPC) client request. The actual calibration parameters are calculated during this procedure based on the received request. In the end, the result is converted to the integer representation and the result message, the result of RPC, is formed.

Finally, the procedure **tune** transfers the calibration result from the client to the device. As with the request in the procedure **update**, it is needed to conduct the transmission over a reliable channel with “at least once” semantics as well.

### 3.1. The Distributed Self-Calibration of the Linear Compensation

The incremental calculation of the covariance matrix S commences by the calculation of the value of the auxiliary vector x from Equation (6), utilizing the procedure given in Table 1, where ADC_x is the result of the A/D conversion of the signal $u_{x}$ in the first measurement bridge of the MR sensor, ADC_y is the result of the A/D conversion of the signal $u_{y}$ in the second measurement bridge of the MR sensor, and ADC0 is the constant corresponding to the half range of the A/D conversion.

The integer representation of the auxiliary vector x obtained in the described way is now used for the calculation of the increment of the covariance matrix S. Since the matrix has only 15 different values, one of them being constant 1, it is sufficient to incrementally calculate values of 14 fields, as shown in Table 2.

The procedure **acquire** can be cancelled before its end, if the set speed becomes less than the highest allowed or if the motor speed observer points out that a significant change of the load happened. In that case, the procedure **acquire** can be restarted once the conditions are fulfilled but not before the predefined timeout period, needed for the transient processes to finish, elapses.

The procedure ends when the shaft finishes a revolution; knowing that the shaft rotates at a constant speed, this corresponds to a constant number of samples. Then, in the procedure **update**, the request for the calibration message is formed, in accordance with Table 3, by combining the unique identifying number of the device, the sequential request number, and the fields of Table 2 describing the covariance matrix S. Since the optimality criterion Equation (8) depends on the sequence of measurements of the input signal u[i] through the covariance matrix S only, the message formed in this way contains all the needed data.

The procedure **evaluate** is executed as a part of the background process on the application server; so, the computational complexity is not critical. It is activated by the received request in the procedure **update**, but the execution is postponed until the processor time is available. The calibration parameters are calculated in this step by solving the matrix system Equation (20) and by the application of Equations (23) and (24) to the obtained result. Finally, the result is converted to integer representation, as in Table 4.

In the end, the request for tuning a message is formed in the procedure **tune**, according to Table 5, by combining a unique identifying number of the device, sequential request number, and the fields from Table 4 describing the calibration result.

For a practical application, it is desirable, not necessary though, to serialize the binary fields in the messages from Table 3 and Table 5 in the network order, that is, in the order they are listed in Table 2 and Table 4, respectively, and in MSB (most significant byte) format.

### 3.2. Application of the LIN Protocol

The LIN (local interconnect network) is a simple communication system and it is widely used for connecting intelligent sensors and actuators with the ECU (electronic control unit) in the automotive industry, due to its low cost and simplicity [22].

Unfortunately, the LIN transport protocol is primarily intended for the realization of the diagnostics’ services and is unsuitable for a general case application. At the same time, the distinctly limited physical address space of the LIN protocol (64 addresses) leaves no possibility for the allocation of an additional pair of physical addresses necessary for the realization of an independent transport communication channel. Additionally, the master/slave transfer model cannot be directly applied to the distributed calibration method shown in Figure 3. In order to overcome this, a data concentrator, a service within the LIN master node, with the role of an agent between the device and the client, was introduced in the realization of the method. The communication between the concentrator and the device is realized by the utilization of the standard UDS (unified diagnostic services) diagnostics’ protocol at the session layer, with the monitoring of sequential request number through the data structures described in Table 3 and Table 5. The OSI model of the interconnection of the device and the concentrator by means of the LIN protocol is shown in Table 6.

**Figure 4:**
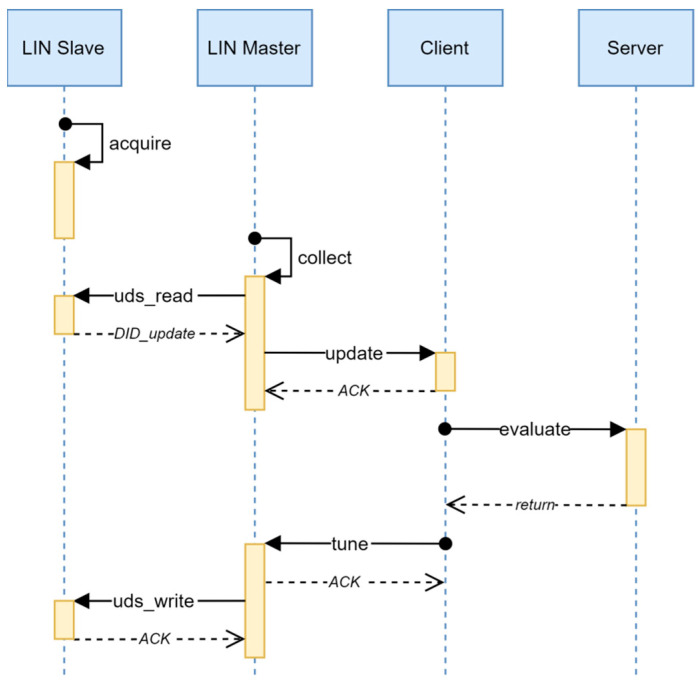
Diagram of distributed calibration method for LIN protocol.

The two different UDS diagnostic identifiers (DID) are employed for communication between the device and the concentrator. The transfer of the calibration request message is realized by means of the standard UDS service 0x22 (read data by identifier), with the first diagnostic identifier (DID_update), and the diagnostic data structure described in Table 3. Similarly, the transfer of the tuning request message is realized by means of the standard UDS service 0x2E (write data by identifier) but with another diagnostic identifier (DID_tune) and the diagnostic data structure described in Table 5. The time diagram of the distributed calibration method adapted to the LIN protocol is shown in Figure 4.

Regarding the distributed self-calibration method shown in Figure 3, the method adapted to the LIN protocol contains an additional procedure **collect** as well as the additional steps in the procedure **tune**. The procedure **collect** is executed periodically on the LIN Master node and it is responsible for the collection of the calibration requests from the LIN slave node by reading the DID_update diagnostic data. If the sequential number of the collected request increases comparing to the sequential number of the last processed request, the new request is being processed by the call of the procedure **update**.

During the procedure **tune**, the received calibration result is additionally transferred to the LIN slave node by writing DID_tune diagnostic data. Again, for the sake of the savings of the bandwidth, this step can be skipped if the sequential number of the last received calibration request in the procedure **tune** is less than or equal to the sequential number of the last transferred calibration result.

### 3.3. Application of the CAN Protocol

The CAN (controller area network) bus is a reliable network system, suited for automotive applications [23]. Its network and transport layer are described in the CAN TP standard ISO 15765 [24]. The reference OSI model of the interconnection of the device and the client by means of CAN protocol is shown in Table 7.

In this application, the transport protocol utilizes two physical addresses from 29-bit address space, which identify a message. The first physical address is used when **update** message from Table 3 is transferred, whereas the second physical address is used for the transfer of **tune** message from Table 5.

The application of the ISO standard protocols in the second group of the OSI layers provides for the utilization of the standard communication equipment and comes down to the configuration of two standard transport channels. This is of utmost importance for usage in an automotive communication network where there are usually several physical and logical buses connected by standard network gateways.

### 3.4. Application of the SOME/IP Protocol

The SOME/IP (service-oriented middleware over IP) is a client–server communication protocol, developed for the realization of advanced systems in the automotive industry. The protocol describes the first group of OSI layers only; the standard TCP/IP or UDP/IP protocol stacks are usually used for the second group of OSI layers [25]. The SOME/IP protocol itself specifies a method for remote procedure calling (remote procedure call, RPC).

The reference OSI model of the interconnection of the client and the application server by means of SOME/IP protocol is shown in Table 8.

The communication between the client and the server is realized by means of a single RPC call, **evaluate**, whose argument is the calibration request message from Table 3; the returned value contains the calibration result from Table 4. Since the needed communication capacity is negligible compared to the physical layer capacity, error recovery is obtained by a simple call repetition.

## 4. Experimental Results

**Figure 5:**
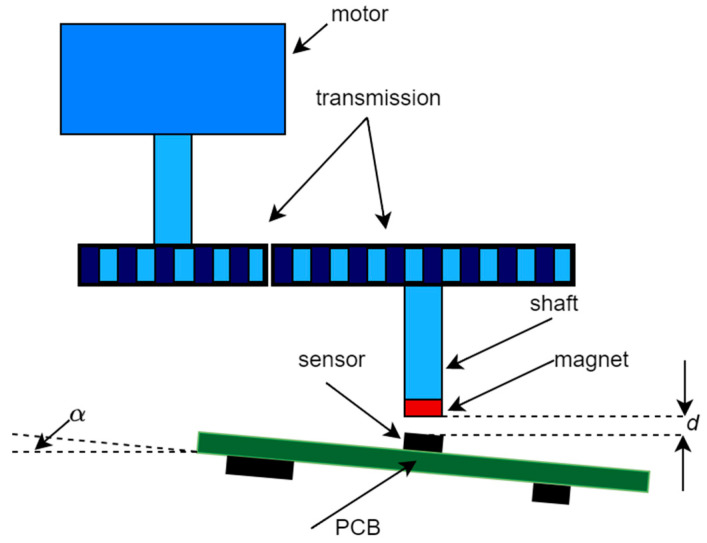
Mechanical diagram of experimental setup.

The proposed method was realized and experimentally verified in an experimental setup, whose principal mechanical diagram is presented in Figure 5. A low-power (12 V, 250 mA) reluctance motor with an 18/149 transmission system and angular position AMR sensor (APS00B [26]) was used. The magnet (radially magnetized, 1 mm thick SmCo washer, with inner and outer diameters of 5 mm and 11 mm, respectively, and nominal magnetic induction of 200 mT) was placed at a relatively large distance (d≈2 mm) from the MR sensor; so, the intensity of the magnetic field was near the lower boundary of the used sensor range, corresponding to realistic circumstances, that, is the situation when the MR sensor and the magnet are placed in hermetically separated spaces. The sensor was mounted on a PCB comprising the electronic subsystem; the PCB could be inclined for an angle α in order to misalign the magnet and the sensor.

**Figure 6:**
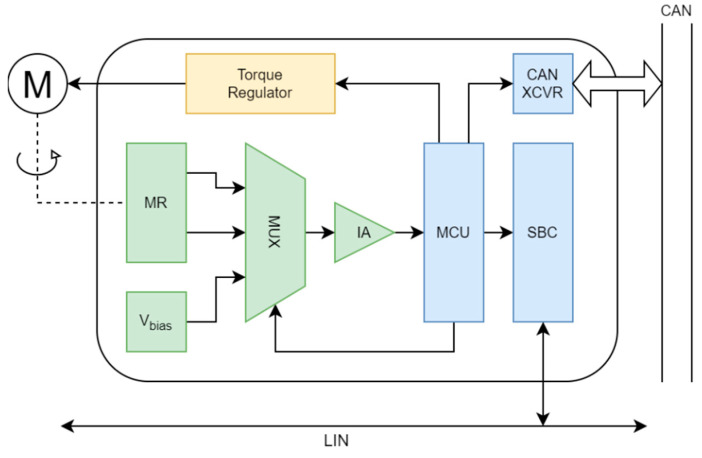
Block diagram of measurement device.

The measurement device, which drives the motor and reads out the sensor, was purposely built for the needs of the research of the advanced methods of the signal processing within the servo systems in the automotive industry; its block diagram is shown in Figure 6. An 8-bit MCU with the integrated CAN and LIN interfaces, suitable for automotive use, was employed (ATMEGA64M1 [16]). The motor is driven through an integrated vector control torque regulator (DVR8825 [27]) controlled by the analog reference inputs, whereas the position and speed regulators are realized within the MCU program. The analog measurement interface consists of an analog multiplexer (MUX) for the selection of the measurement input and an instrumentation amplifier (IA) for the amplification of the input before A/D conversion. This way, both MR measurement bridges use the same IA, so its gain error does not affect the measured angle. In the third MUX position, when a bias voltage equal to one-half of the reference voltage is selected, the analog interface offset is measured in order to compensate for it independently from the self-calibration procedure. The system also utilizes a system basis chip (SBC, ATA6622C [28]) containing a power supply controller, a watchdog, and a communication interface for the connection with an automotive platform over the LIN interface. In order to prove the concept, the measurement device was connected to a commercial AUTOSAR-AP server running the self-calibration service. Finally, there was a CAN transceiver in the system, used for sending data to a remote computer for off-line analysis.

It should be noted that the described mechanical construction allowed for the qualitative determination of the relative position of the sensor and the magnet only. Thus, the experiment was repeated in three basic configurations. The axes of the magnet and the sensor “almost” matched, so there was approximately no (just an offset) inclination of the sensor in the first configuration. A permanent field of magnetic interference was added in the second configuration. In the end, in the third configuration, the angle between the axes of the magnet and the sensor, that is, the sensor inclination, was chosen to be the largest possible so the magnetic field intensity in the sensor plane remained within the nominal operational range of the sensor regardless of the angular position (approximately 15°). The purpose-built diagnostic interface within the measurement device was used for direct collection of A/D conversion results from within an interrupt routine, thus providing for direct comparison of various methods.

**Figure 7:**
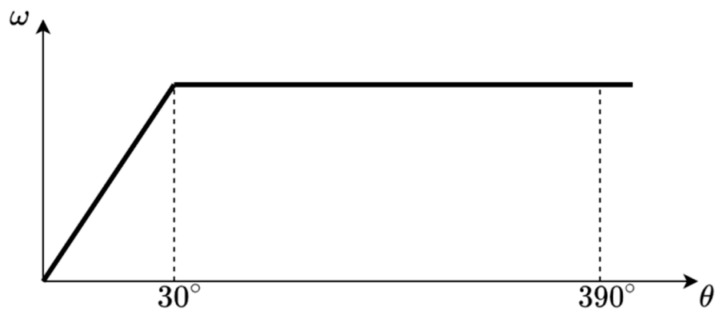
The speed profile of the servo system in a function of the relative angular movement.

Figure 7 presents the speed profile of the servo system in the function of the angular movement relative to the initial position when the set point was abruptly changed. The speedup ramp was completed and the maximal rotational speed was reached when the shaft rotated 30° from the initial position. From then on, the shaft continued the rotation with the constant rotational speed until reaching 30° from the set target, where the slowdown ramp started. Consequently, the data for the self-calibration were available once the shaft rotated 390° from the initial position, provided that the set-point difference was at least 420°.

The sensor sampling period of 10 ms represented the base sampling time for the whole system. When rotating at a maximal rotational speed, the shaft rotation period of 3.97 s was equivalent to 397 sample points. The duration of the client/server communication was 210 ms (LIN scheduler table configured duration); so, the total calibration procedure duration, including the speedup ramp, was 5.38 s.

**Figure 8:**
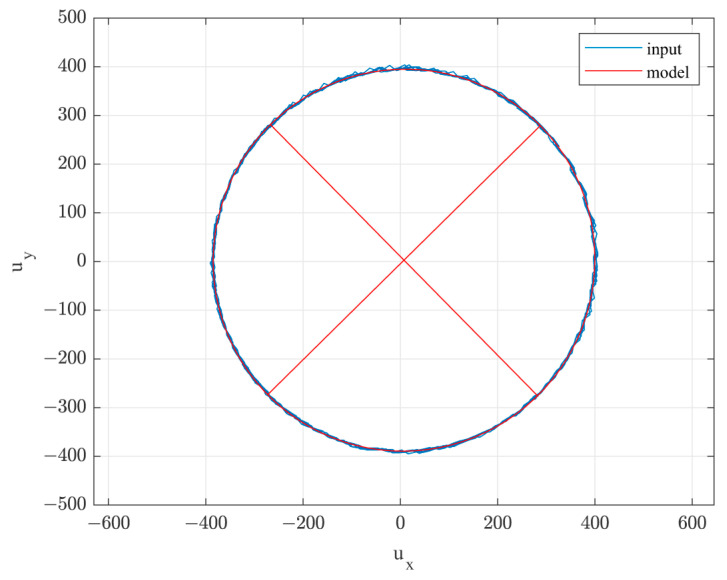
The input signal trace and the identified MR sensor model.

The trace of the input signal u in the experiment with the third configuration is shown in Figure 8. Despite extremely unfavorable experiment conditions, the trace showed exceptional matching to the model. The misalignment of the axis and the permanent interference field caused the subtle translational movement of the ellipse only, whereas the remaining model parameters were practically identical in all the experiments. Such a result clearly confirmed the validity of the introduction of independent compensation of the magnetic field vector measurement.

**Figure 9:**
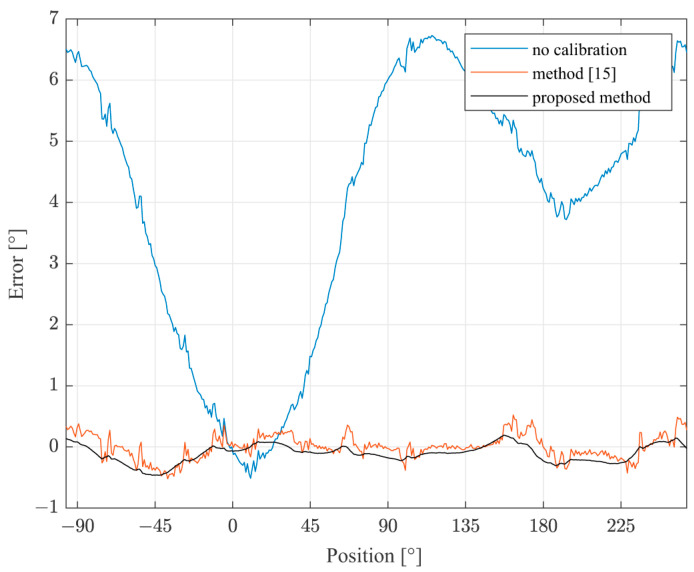
The deviation of the angle from the reference before and after the correction, with the inclined sensor.

Figure 9 presents the deviation of the magnetic angle from the reference angle in the third configuration experiment, without the calibration (blue), after the calibration using the method from [15] (red) and after the calibration using the proposed method (black). The largest error occurred when the distance between the magnet and the sensor was the largest, when the intensity of the magnetic field was at the sensor sensitivity boundary. Without the compensation, the error boundary reached 7°, unacceptable for a majority of the applications. However, the error boundary was lowered to only 0.5° by the utilization of the proposed method.

**Figure 10:**
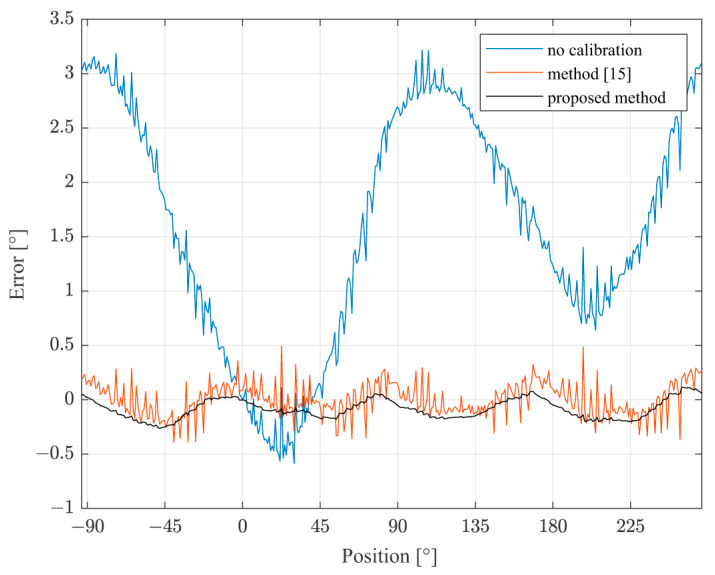
The deviation of the angle from the reference before and after the correction, with the uninclined sensor.

As a comparison, when the experiment was repeated in an identical manner, except that the sensor was approximately not inclined, the result was significantly different only in the direction of the offset inclination, as shown in Figure 10.

Three additional calibration methods were selected for comparison, the offset compensation method based on [12], the linear compensation method based on models from [9] and [11], and the method from [15]. The methods from [10] and [13] were not considered, as they require a specific sensor, while the method from [14] requires magnetic barriers. Similarly, methods employing multilayered neural networks were not considered as the experimental apparatus lacked the mechanical long-term stability required to produce the relevant training set. Only the proposed method was suitable for real-time execution, while all the remaining methods were run off-line using the collected raw sensor data. Input data had to be additionally filtered for the offset calibration method application in order to reduce the noise sensitivity to an acceptable level. The measurement results are presented in Table 9, Table 10 and Table 11 for the first, second, and third experimental configurations, respectively.

It was noticeable that mechanical tolerances substantially influenced the measurement error variance prior to the compensation, which was significant in the case of good mechanical alignment as well. However, the application of the proposed method reduced the mean squared error for two orders of magnitude, bringing it down to the level of the sensor resolution. No significant influence of the mechanical configuration to the error variance was noted after the correction, apart for extreme cases, pointing out a good suppression of the apparatus’ mechanical tolerances.

The estimated absolute of the measurement error was reduced by an order of magnitude, comparable to the modern EOL calibration procedures.

## 5. Discussion

The presented results confirm the applicability of the proposed method for the self-calibration of the magnetoresistive angular position sensor within a servo system. Since the servo system itself behaves as a low-pass filter, the stochastic measurement errors are being additionally suppressed, and the expected boundary of the positioning error was better than the estimated boundary of the measurement error.

The comparison with the method from [15] points out that the proposed method achieves significantly better measurement accuracy, with the error being 30% to 60% lower. Partially, this improvement is due to the more complex linear self-calibration algorithm which is, because of the distributed realization on the application server, not limited by the processing power of the measurement device. Such an algorithm also enabled the application of the improved harmonic calibration algorithm. Finally, it was shown that the introduction of the speed observer significantly reduced the influence of the measurement noise and the electromagnetic interference to the measurement error, which was especially noticeable in the experiment with the uninclined sensor (Figure 10) where the best improvement was noted at the same time.

The proposed method required only a modest input data set, collected during one full cycle of the shaft rotation at maximal speed. In comparison, the gradient descent algorithms, as in [9] and [11], require considerably more input data, typically collected during 50 to 100 full shaft rotations. It should be noted that there are typically only five cycles of shaft rotation per driving session for a general vehicle. This makes the proposed method uniquely suitable for automotive applications, where the set-point changes are relatively infrequent compared to the parameter drifts caused by extreme mechanical and temperature stresses.

## 6. Conclusions

In this paper, a method for the distributed self-calibration of a magnetoresistive angular position sensor within a servo system was presented. The accuracy of the angular position measurement by means of the MR sensors was unfavorably influenced by mechanical tolerances of the measurement system, especially the alignment deviations of the sensor axis, the magnet axis, and the rotation axis. For that reason, the system for the compensation of the measurement error was integrated within the control structure of the servo system. The system consisted of the linear compensation of the magnetic field vector measurement error, followed by the application of the harmonic compensation of angular position measurement error.

The proposed self-calibration method for the system for the determination of angular position utilized the data collected during the movement at the highest allowed speed for the identification of the measurement process model parameters. The identification was performed at the times when the shaft finished a revolution. The method of minimizing the sum of algebraic distances of the sensor readings and the parametrized model was employed for the identification of parameters of the linear compensation, whereas the average rotation speed was used as a high-accuracy reference for the identification of the harmonic compensation parameters.

The proposed distributed calibration method utilized the spare capacity of the existing communication infrastructure exclusively, with no significant influence over the speed and the delay of pre-existing services. It was accomplished by the splitting of the method into four asynchronous procedures, each step being executed only when the required resources were available. An appropriate application protocol was developed, and the possibility of an application with the existing transport protocols was pointed out. Finally, a reliable, UDS-based, and LIN-applicable transport protocol was shown, as well as a corresponding concentrator for the AUTOSAR-CP system.

The proposed method was practically realized on an embedded device, constructed in accordance with the needs of the automotive industry. Both occasional parameters’ identification and the distributed realization simplified the realization, despite considerable computing complexity of the proposed method. It was shown that the application of the proposed self-calibration method increased measurement accuracy for an order of magnitude. The achieved measurement uncertainty was less than 0.5° and is comparable to the results of the modern EOL calibration procedures. In addition, a fast convergence was achieved, lasting only 5.38 s, making it the only self-calibration procedures that could be completed during a typical driving session.

A markedly good match between the experimental results and the theoretical model opens several potential directions for further research. The results suggest that the self-calibration of the linear compensation could be executed independently from the rotation speed, instead of when the motor shaft rotates at the highest speed. Additionally, the application of a gradient algorithm for the tuning of harmonic compensation could be considered, in order to further reduce the needed computing capacity.

The results from this paper could be extended to the self-calibration of the Hall effect angular position sensors as well, in the applications where the magnetic field intensity is not a limiting factor. Since the Hall sensors possess a better linearity than MR sensors, the linear compensation is less important compared to the MR sensors. However, considering that the primary source of nonlinear effects is not the sensor itself but the misalignment of the magnet, the harmonic compensation would be equally efficient. Therefore, it is expected that the procedure would be applicable to the Hall sensors as well.

## Figures and Tables

**Table 1:** Calculations of the auxiliary vector.

Field	Data Type	Value	Description
in_x	Int16	ADC_x − ADC0	Signal $u_{x}\left[i\right]$
in_y	Int16	ADC_y − ADC0	Signal $u_{y}\left[i\right]$
in_x2	Int32	in_x × in_x	Signal ${\left(u_{x}\left[i\right]\right)}^{2}$
in_y2	Int32	in_y × in_y	Signal ${\left(u_{y}\left[i\right]\right)}^{2}$
in_xy	Int32	in_x × in_y	Signal $u_{x}\left[i\right]\cdot u_{y}\left[i\right]$

**Table 2:** Calculations of the covariance matrix.

Field	Data Type	Increment	Description
S_x4	Int64	in_x2 × in_x2	Sum ${\left(u_{x}\left[i\right]\right)}^{4}$
S_y4	Int64	in_y2 × in_y2	Sum ${\left(u_{y}\left[i\right]\right)}^{4}$
S_x3y	Int64	in_x2 × in_xy	Sum ${\left(u_{x}\left[i\right]\right)}^{3}\cdot u_{y}\left[i\right]$
S_y3x	Int64	in_y2 × in_xy	Sum ${\left(u_{y}\left[i\right]\right)}^{3}\cdot u_{x}\left[i\right]$
S_x2y2	Int64	in_y2 × in_x2	Sum ${\left(u_{x}\left[i\right]\right)}^{2}\cdot {\left(u_{y}\left[i\right]\right)}^{2}$
S_x3	Int64	in_x2 × in_x	Sum ${\left(u_{x}\left[i\right]\right)}^{3}$
S_y3	Int64	in_y2 × in_y	Sum ${\left(u_{y}\left[i\right]\right)}^{3}$
S_x2y	Int64	in_x2 × in_y	Sum ${\left(u_{x}\left[i\right]\right)}^{2}\cdot u_{y}\left[i\right]$
S_y2x	Int64	in_y2 × in_x	Sum ${\left(u_{y}\left[i\right]\right)}^{2}\cdot u_{x}\left[i\right]$
S_x2	Int32	in_x2	Sum ${\left(u_{x}\left[i\right]\right)}^{2}$
S_y2	Int32	in_y2	Sum ${\left(u_{y}\left[i\right]\right)}^{2}$
S_xy	Int32	in_xy	Sum $u_{x}\left[i\right]\cdot u_{y}\left[i\right]$
S_x	Int32	in_x	Sum $u_{x}\left[i\right]$
S_y	Int32	in_y	Sum $u_{y}\left[i\right]$

**Table 3:** Request for calibration message.

Field	Data Type	Description
Device	UInt32	Unique identifying number of the device
Sequence	UInt32	Sequential request number
S_*	Byte[92]	Fields describing covariance matrix S from Table 2

**Table 4:** Calibration result.

Field	Data Type	Matrix	Description
R_Ox	Int16	o	Constant offset along *x*-axis, $o_{x}$
R_Oy	Int16	o	Constant offset along *y*-axis, $o_{y}$
R_G11	Int16	$G^{-1}$	Compensation matrix, element (1,1)
R_G22	Int16	$G^{-1}$	Compensation matrix, element (2,2)
R_G12	Int16	$G^{-1}$	Compensation matrix, element (1,2)

**Table 5:** Request for tuning message.

Field	Data Type	Description
Device	UInt32	Unique identifying number of the device
Sequence	UInt32	Sequential request number
R_*	Byte[10]	Fields (calibration results) from Table 4

**Table 6:** OSI model of interconnection of the device and the concentrator by means of the LIN protocol.

Layer	Protocol
Application	Section 2, Figure 3
Presentation	Section 3.1
Session	UDS, Table 3 and Table 5
Transport	ISO 17987-2
Network	ISO 17987-2
Data Link	ISO 17987-3
Physical	ISO 17987-4

**Table 7:** OSI model of interconnection of the device and the client by means of the CAN protocol.

Layer	Protocol
Application	Section 2, Figure 3
Presentation	Section 3.1
Session	Table 3 and Table 5
Transport	ISO 15675-2
Network	ISO 15675-2
Data Link	ISO 11898
Physical	ISO 11898

**Table 8:** OSI model of interconnection of the client and the server by means of SOME/IP protocol.

Layer	Protocol
Application	Section 2, Figure 3
Presentation	Section 3.1
Session	SOME/IP: RPC interface
Transport	UDP
Network	IP
Data Link	IEEE Ethernet MAC + VLAN (802.1Q)
Physical	Automotive Ethernet. 100/1000Base-T1

**Table 9:** Experimental results, with aligned axes and uninclined sensor.

Method	Max	Mean	Variance	MSE
No Compensation	3.1618	−1.5327	0.8844	3.2334
Offset Compensation	2.5792	−1.5208	0.8844	2.7734
Linear Compensation	2.2340	−0.0164	1.1390	1.1391
Method from [15]	0.5777	−0.0049	0.0279	0.0279
Proposed Method	0.4102	0.0815	0.0100	0.0167

**Table 10:** Experimental results, with aligned axes, uninclined sensor, and permanent interfering field.

Method	Max	Mean	Variance	MSE
No Compensation	3.7734	−1.2207	1.7774	3.2646
Offset Compensation	3.0553	−1.2069	0.8389	2.3939
Linear Compensation	2.8736	−0.1358	2.6262	2.6402
Method from [15]	0.4073	−0.0014	0.0179	0.0179
Proposed Method	0.1593	−0.0091	0.0047	0.0048

**Table 11:** Experimental results, with substantially unaligned axes and inclined sensor.

Method	Max	Mean	Variance	MSE
No Compensation	7.0427	−3.9452	4.2433	19.808
Offset Compensation	6.2475	−3.9283	3.2137	18.645
Linear Compensation	4.5289	−0.0087	4.9284	4.9282
Method from [15]	0.7501	−0.0070	0.0369	0.0369
Proposed Method	0.4973	0.0969	0.0218	0.0312
